# Hypoglossal nerve stimulation versus positive airway pressure therapy for obstructive sleep apnea

**DOI:** 10.1007/s11325-022-02663-6

**Published:** 2022-07-02

**Authors:** Clemens Heiser, Armin Steffen, Patrick J. Strollo, Claire Giaie-Miniet, Olivier M. Vanderveken, Benedikt Hofauer

**Affiliations:** 1 Department of Otorhinolaryngology, Head and Neck Surgery, Technical University Munich, Ismaninger Str. 22, 81675 Munich, Germany; 2 Translational Neurosciences, Faculty of Medicine and Health Sciences, University of Antwerp, Antwerp, Belgium; 3 University of Lubeck, Luebeck, Germany; 4 Department of Otolaryngology, University of Pittsburgh, Pittsburgh, PA USA; 5 Department of ENT, Head and Neck Surgery, Antwerp University Hospital, Edegem, Belgium

**Keywords:** Hypoglossal nerve stimulation, Obstructive sleep apnea, Positive airway pressure, Sleepiness, Upper airway stimulation

## Abstract

**Purpose:**

Hypoglossal nerve stimulation (HNS) has been shown to treat obstructive sleep apnea (OSA) effectively. The aim of this study was to compare HNS with positive airway pressure (PAP) treatment regarding outcome parameters: (1) sleepiness, (2) apnea–hypopnea index (AHI), and (3) effectiveness.

**Methods:**

Propensity score matching with nearest neighbor algorithm was used to compare outcomes of HNS and PAP therapy in a real-world setting. Data were collected at baseline and 12 months after initiating OSA treatment including demographics, Epworth Sleepiness Scale (ESS), AHI, and objective adherence data. To account for overall treatment efficacy, the mean disease alleviation (MDA) was calculated.

**Results:**

Of 227 patients who received treatment consecutively, 126 could be matched 1:1 with regard to age, body mass index, and AHI. After matching, no statistically significant differences between the groups were found. A clinically important symptom improvement was seen at 12 months in both cohorts, though there was a greater difference in ESS improvement in patients treated with HNS (8.0 ± 5.1 points vs. 3.9 ± 6.8 points; *p* = 0.042). In both groups, mean posttreatment AHI was significantly reduced (HNS: 8.1 ± 6.3/h; PAP: 6.6 ± 8.0/h; *p* < 0.001). Adherence after 12 months among patients treated with HNS was higher than in those receiving PAP therapy (5.0 ± 2.6 h/night; 4.0 ± 2.1 h/night) but not with statistical significance. Overall effectiveness calculated with the MDA was 59% in patients treated with HNS compared to 51% receiving PAP.

**Conclusion:**

Patients treated with HNS therapy had significantly greater improvements in daytime sleepiness compared to PAP therapy, while the mean reduction of AHI and overall effectiveness were comparable for both treatments.

**Trial registration:**

ClinicalTrial.gov Identifier: NCT03756805.

## Introduction

Obstructive sleep apnea (OSA) is one of the most prevalent chronic conditions [1]. The disease is characterized by partial or total collapse of the pharyngeal airway and is associated with excessive daytime sleepiness, neurocognitive impairment, increased risk of cardiovascular diseases, and a diminished quality of life [2]. As standard therapy, positive airway pressure (PAP) via a nasal or oro-nasal mask is available in many healthcare systems. For mild and moderate sleep apnea, oral appliances that advance the mandible and open the posterior airway space are considered equivalent to PAP therapy [3]. Though highly efficacious in maintaining acute airway patency, PAP therapy’s long-term effectiveness is often diminished due to low acceptance and adherence [4]. Different strategies, such as patient education and treatment of coexisting sleep disorders, could favorably impact PAP usage and lead to lower discontinuation rates [5, 6].

In addition to a reliable diagnosis in the sleep laboratory, predictors of adherence include the severity of the illness, daytime sleepiness, and the usage pattern during the first week of PAP therapy [7, 8]. Improvements of daytime sleepiness, performance, quality of life, and blood pressure can also contribute significantly to longterm adherence. Nevertheless, up to 50% of patients prescribed PAP therapy will discontinue therapy in the first 12 months [4]. To ensure sufficient OSA control, annual assessments of treatment response and adherence are recommended to avoid negative consequences from un- or undertreated OSA [9].

In addition to objective measurements such as the apnea–hypopnea Index (AHI), symptoms such as daytime sleepiness are critical to assess treatment effectiveness. A reliable and valid tool to measure sleepiness is the Epworth Sleepiness Scale (ESS), which is one of the most important instruments to evaluate quality of life in patients with OSA [9]. Sleepiness is a major symptom in OSA patients and is independently associated with a worse prognosis for mortality and cardiovascular diseases. A recently published cluster analysis of OSA examined endotypes of participants relative to self-reported sleep symptoms [10]. Three different endotypes were identified: *excessive sleepiness*, *disturbed sleep*, and *non-sleepiness*. A current systematic review of the effect of PAP therapy for OSA in elderly participants found that improvements in sleepiness were associated with improvements of mood, cognition, and quality of life [11].

Hypoglossal nerve stimulation (HNS) has been developed as an alternative treatment for OSA patients with intolerance to PAP therapy. It was successfully implemented in the routine OSA care pathways in Europe and North America [12–15]. Sustainable beneficial effects on respiratory parameters, sleep architecture, and arousals have been demonstrated with HNS therapy [16–18]. Acceptance of this new treatment by patients has been good, with reported adherence of more than 5 h per night on average and significant improvements of sleepiness and alertness [19–22]. Effects have been shown to be sustainable up to 5 years of follow-up [13, 15]. HNS has been demonstrated to be more effective than classical surgical interventions such as soft palate or tongue base surgery [23, 24, 39, 40]. In some patients, HNS can even be “curative” in normalizing the AHI to < 5 events per hour [14].

To properly evaluate the overall effectiveness in chronic conditions like OSA, it is important to consider not only the acute efficacy but also the long-term adherence to treatment. To achieve this, the concept of mean disease alleviation (MDA) has been introduced into respiratory sleep medicine [25]. The MDA is defined as the product of adjusted adherence and treatment efficacy, divided by 100 and expressed in percentage. The resulting value then describes the overall therapeutic effectiveness of treatment over the evaluated time period.

The aim of this study was to compare HNS with PAP therapy on the patient-relevant outcome of self-reported sleepiness. A secondary endpoint included differences of AHI improvements as well as therapy adherence between both treatments. To compare the cohorts who received the two treatments, we performed propensity score matching to assemble well-balanced groups based on the variables age, BMI, and baseline AHI. The calculated clinical effectiveness was assessed utilizing MDA.

## Methods

This multicenter prospective clinical trial included patients who received an HNS system (Inspire Medical Systems, Golden Valley, Minnesota, USA) or PAP therapy for treatment of OSA. The study was approved by the local ethics committee (number 80/18 S) and was registered as NCT03756805 on clinicaltrials.gov.

The study population consisted of patients who presented with a diagnosis of OSA to the Departments of Otorhinolaryngology at the University Hospitals Munich, Germany (Department of Otorhinolaryngology/Head and Neck Surgery in Klinikum rechts der Isar, Technical University of Munich) and Luebeck, Germany (Department of Otorhinolaryngology, Phoniatrics and Pediatric Audiology, University Hospitals of Schleswig–Holstein, Campus Luebeck) and were treated with either PAP or HNS therapy.

Treatments were initiated between June 2014 and December 2018. Participants were enrolled consecutively from clinical practice cohorts, if they had been using the same treatment exclusively for at least 12 months and if they had pathological sleepiness, with an ESS value of ≥ 11. Subjects were enrolled independently of therapy response status. Exclusion criteria were a BMI ≥ 35 kg/m^2^ or an AHI < 15 or > 65 and/or > 25% central apneas on baseline sleep study. In addition, patients with anatomical abnormalities, defined as tonsil grade > 3 per Friedman classification 3; lingual tonsil hypertrophy > 3; severe retrognathia; and Friedman tongue position > IIb, were excluded since only very few subjects for each condition were available for analysis in our clinics.

PAP therapy was initiated as first-line treatment in patients with a new diagnosis of OSA, in accordance with German guidelines for treatment of sleep-disordered breathing [26].

Patients received an HNS system as second-line treatment after failure or intolerance of PAP treatment. As part of the screening process, HNS patients had to undergo drug-induced sleep endoscopy to rule out soft palate complete concentric collapse. Surgical implantation of the HNS system was conducted as described previously [27, 28].

In both groups, data were collected at baseline and 12-month follow-up. Demographic and physiological information were collected and the ESS questionnaire was utilized to evaluate self-reported sleepiness [29]. In line with reimbursement directives for sleep tests in Germany, a polysomnography (PSG) was used for baseline assessment of OSA, and a home sleep test (HST) was performed to evaluate treatment effects at follow-up. For both groups, the clinical pathway was the same to depict routine clinical work. Objective outcomes were manually scored according to the American Academy of Sleep Medicine criteria using hypopnea scoring with at least 30% nasal pressure signal reduction and 4% oxygen desaturation [30]. At 12-month follow-up, no further device adjustments were allowed to evaluate the treatment effects properly.

In the PAP cohort, airflow was measured with an outlet between tube and mask at 12-month follow-up. The AHI was calculated from HST data. PAP usage time was objectively measured in hours of use per night using the built-in counter in the PAP device at 12-month follow-up.

For the HNS cohort, the postoperative therapy initiation and fine-tuning were conducted in accordance with the recommendations as published previously [31]. Treatment usage time was collected in hours of use per night, derived from interrogation of the implantable pulse generator at 12-month follow-up.

The primary endpoint of this study was the assessment of the effect of the two treatment alternatives on subjective sleepiness evaluated with the ESS.

### Statistical analysis

Version 26.0 of the Statistical Package for the Social Sciences software (SPSS, Chicago, Illinois, USA) was used. Descriptive statistics were calculated for demographic variables, and Student’s *t* tests were used for continuous and *χ*^2^ tests for categorical variables. When necessary, nonparametric alternatives (Mann–Whitney *U* test, Fisher exact test) were applied. The Kolmogorov–Smirnov test was used to evaluate distribution of data, and data were considered normally distributed if *p* > 0.05. Outcome measures of AHI and ESS from 12-month follow-up were compared to baseline measurement. Participants with incomplete data sheets on age, gender, BMI, AHI, and ESS were excluded from the analysis.

**Fig. 1:**
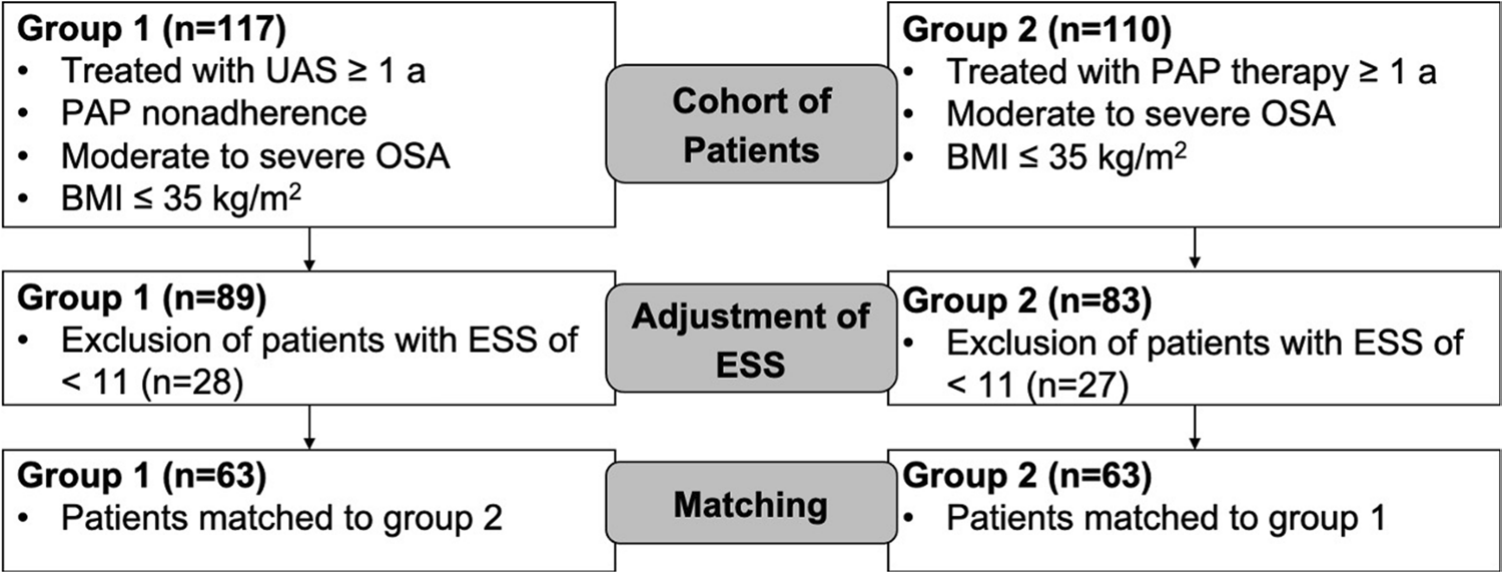
Flowchart of propensity score matching, which was used to assemble well-balanced groups based on the variables age, BMI and baseline AHI. ESS, Epworth Sleepiness Scale; HNS, hypoglossal nerve stimulation; PAP, positive airway pressure; ESS, Epworth Sleepiness Scale; BMI, body mass index; AHI, apnea–hypopnea index

To assemble well-balanced groups, propensity score matching was performed, based on the variables age, BMI, and baseline AHI (Fig. 1). These parameters were chosen as variables for matching due to their independent association with outcomes of OSA treatments. Participants from the HNS group were matched 1:1 to participants in the PAP group according to the propensity score using the nearest neighbor-matching algorithm (Fig. 1). The caliper value was set to 0.4 logit propensity score standard deviations and *p*-values ≤ 0.05 were considered statistically significant.

To assess the overall clinical effectiveness of the two treatments, the MDA was calculated as a combined function of efficacy and adherence [25]. The MDA is defined as the product of adjusted adherence and therapeutic efficacy, defined as reduction in AHI, divided by 100 and expressed in percentage points. Optimal adherence was operationally defined as 6.5 h/night, as this would equal to full adherence for the average sleep duration in Europe [41]. The residual AHI can thus be calculated as the product of baseline AHI and (100 − MDA)/100 [25].

## Results

A total of 227 participants were consecutively enrolled between June 2014 and December 2018 and were eligible for further analysis: group 1 consisted of 117 patients treated with HNS therapy and group 2 of 110 patients who received PAP therapy. After exclusion of subjects with an ESS < 11 and propensity score matching, two homogeneous groups with 63 patients each were constructed (Fig. 1). No statistically significant differences with regard to the pre-defined matching variables were found (age: *p* = 0.985; BMI: *p* = 0.160; baseline AHI: *p* = 0.146). In addition, there were no significant differences for gender, race, and comorbidities (Table 1).

**Table 1:** Comparison of baseline demographic and clinical characteristics for participants treated with HNS and PAP prior to and following propensity score matching. *ESS*, Epworth Sleepiness Scale; *HNS*, hypoglossal nerve stimulation; *PAP*, positive airway pressure; *BMI*, body mass index; *AHI*, apnea–hypopnea index; *SD*, standard deviation

Variable	Prior to propensity score matching				Following propensity score matching			
	HNS cohort	PAP cohort	Standardized difference	*p*-value	HNS cohort	PAP cohort	Standardized difference	*p*-value
Number	117	110			63	63		
Age ± SD (y)	57.8 ± 11.6	57.9 ± 12.4	0.003	0.925	57.3 ± 12.2	57.4 ± 12.5	0.013	0.985
Female	12 (10%)	36 (33%)		< 0.001	7 (11%)	20 (32%)		0.084
Race	115 Caucasians, 2 Blacks	107 Caucasians, 2 Blacks, 1 Asian			61 Caucasians, 2 Blacks	61 Caucasians, 2 Blacks		
Comorbidities								
Arterial hypertension					23/63	22/63		0.500
Diabetes mellitus					7/63	6/63		0.500
Coronary heart disease					2/63	4/63		0.340
Previous surgery for OSA	None				None			
BMI ± SD (kg/m^2^)	29.5 ± 3.7	31.4 ± 5.9	0.384	0.025	29.2 ± 4.1	30.6 ± 5.0	0.303	0.160
Baseline AHI ± SD (n/h)	36.5 ± 14.8	39.6 ± 26.7	0.144	0.538	33.9 ± 15.1	36.8 ± 21.6	0.157	0.146

Among patients treated with PAP, the most used ventilation mode was continuous PAP (68%), followed by automated PAP (19%) and bilevel PAP (S-mode, 13%). Patients with bilevel PAP were using this mode not for complicated sleep-disordered breathing or comorbidities, but to experience lower pressures during exhalation. The mean pressure among PAP users was 7.5 ± 2.6 mbar.

In the HNS group, the most common stimulation mode was bipolar (84%). All patients who received an HNS system had a history of PAP usage, and all underwent different optimization attempts. The most common side effect were mask problems, such as pressure leakage, which lead to therapy discontinuation.

**Fig. 2:**
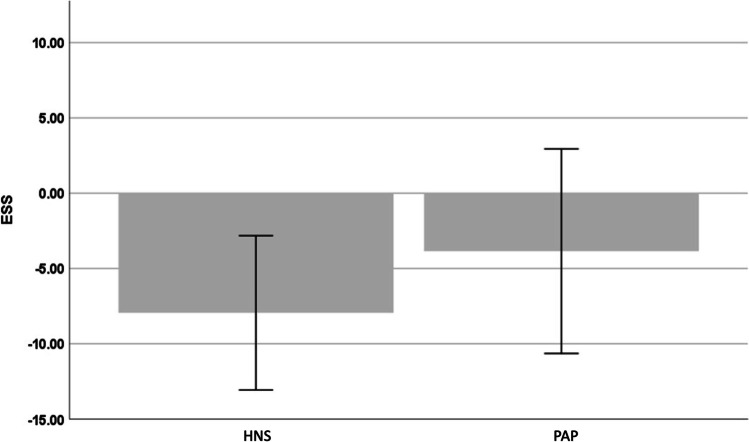
Comparison of mean absolute reduction of ESS in participants with UAS and PAP from baseline to 12-month follow-up (*p* = 0.042). ESS, Epworth Sleepiness Scale; HNS, hypoglossal nerve stimulation; PAP, positive airway pressure

Baseline ESS, as primary outcome of patient-reported sleepiness, was statistically higher in the HNS cohort than in the PAP group (15.4 ± 3.5 vs. 14.6 ± 3.9, Table 2). Sleepiness improved clinically with both treatments and was 7.5 ± 4.7 with HNS therapy versus 10.8 ± 5.6 with PAP treatment at 12-month follow-up. However, the difference of 3.3 points between the two cohorts was not statistically significant (*p* = 0.268). Within-group comparisons of baseline and 12-month follow-up, ESS values showed a significant reduction in the HNS cohort (*p* < 0.001) and a statistically non-significant reduction in the PAP cohort (*p* = 0.056). In absolute values, a reduction of 7.95 ± 5.12 points in the HNS group and 3.86 ± 6.79 points in the PAP group was observed between baseline and 12-month follow-up, which represents a statistically significant difference (*p* = 0.042; Fig. 2).

**Table 2:** Comparison of the baseline and posttreatment values for participants with HNS and PAP and absolute reduction in ESS including *p*-values for comparison between baseline ESS, posttreatment ESS and reduction of ESS between HNS and PAP (HNS vs PAP) and *p*-values for comparison in-between both groups (baseline vs posttreatment). *ESS*, Epworth Sleepiness Scale; *HNS*, hypoglossal nerve stimulation; *PAP*, positive airway pressure; *SD*, standard deviation

Variable	HNS cohort	PAP cohort	*p*-value (UAS vs PAP)
Baseline ESS ± SD	15.4 ± 3.5	14.6 ± 3.9	0.050
12-month follow-up ESS ± SD	7.5 ± 4.7	10.8 ± 5.6	0.268
ESS reduction ± SD	8.0 ± 5.1	3.9 ± 6.8	0.042
*p*-value (baseline vs. 12-month follow-up)	< 0.001	0.056

The usage time was 5.0 ± 2.6 h/night in the HNS cohort and was higher than in the PAP population with 4.0 ± 2.1 h/night without reaching statistical significance (*p* = 0.087; Table 3). There were no differences in recording time of the home sleep test, which was used for therapy efficacy assessment, between the groups.

**Table 3:** Comparison of usage time for participants with HNS and PAP. *SD*, standard deviation; *HNS*, hypoglossal nerve stimulation; *PAP*, positive airway pressure

Variable	HNS cohort	PAP cohort	*p*-value
Usage time ± SD (hours/night)	5.04 ± 2.58	4.03 ± 2.12	0.087

The mean AHI at 12-month follow-up was 6.6 ± 8.0 in the PAP cohort and 8.1 ± 6.3 in the HNS cohort, resulting in a significant reduction in both groups (*p* < 0.001 in both groups). In absolute values, the reduction of 30.9 ± 21.9 observed in the PAP cohort did not differ significantly from 23.0 ± 13.0 recorded in the HNS cohort (*p* = 0.075). At 12-month follow-up, an AHI < 15 was observed in 87% of PAP and 84% of HNS patients, an AHI of < 10 in 81% and 74%, and an AHI of < 5 in 59% and 51% respectively. All observed differences were statistically insignificant (*p* = 0.626; *p* = 0.268; *p* = 0.269).

### Overall clinical effectiveness calculation

**Fig. 3:**
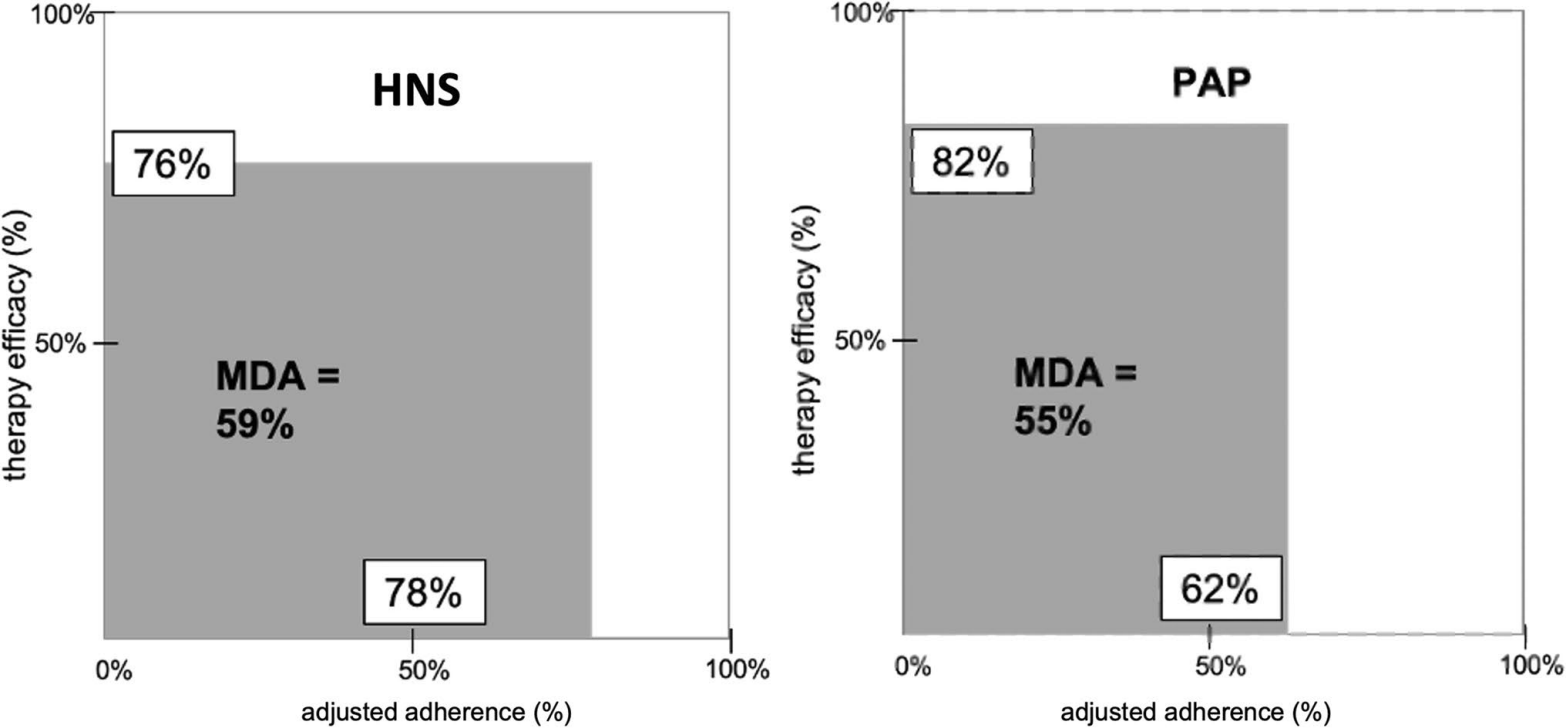
Mean disease alleviation (MDA) for PAP and HNS therapy. MDA is equal to the surface area of the rectangle for which the length is given by the adjusted adherence (usage time/total sleep time), and the height is given by the therapeutic efficacy (AHI baseline minus AHI with therapy applied, expressed in percentage). MDA provides a measure of overall therapeutic effectiveness. PAP, positive airway pressure; HNS, hypoglossal nerve stimulation

Overall clinical effectiveness, considering AHI reduction and therapy adherence at 12 months, was higher with HNS therapy with an MDA of 59%, compared to PAP treatment with an MDA of 51% (Table 4; Fig. 3). The driver of greater overall clinical effectiveness calculated with MDA in patients with HNS treatment was a higher adjusted adherence compared to PAP therapy, with only slightly lower therapeutic efficacy. The residual AHI at 12-month follow-up, calculated from the MDA for both treatments, was 14/h in the HNS group and 18/h in the PAP group.

**Table 4:** Calculating the mean disease alleviation (MDA) and overall remaining AHI for both treatments HNS and PAP. *HNS*, hypoglossal nerve stimulation; *PAP*, positive airway pressure; *AHI*, apnea–hypopnea index

	HNS cohort	PAP cohort
Therapeutic efficacy (%)	76	82
Adjusted adherence (%)	78	62
Mean disease alleviation (MDA) (%)	59	51
Overall remaining AHI (/hour sleep)	14	18

## Discussion

This is the first study to compare HNS therapy to PAP treatment in a homogeneous cohort of matched patients with OSA. Propensity score matching was used to account for parameters that are known to influence outcomes of OSA treatments, such as baseline AHI and body mass index. In the two comparable groups in our study, we could show that HNS therapy is superior in improving daytime sleepiness compared to PAP treatment (ESS reduction 7.95 points versus 3.86 points). The observed degree of reduction with PAP treatment is consistent with results of prior meta-analyses, according to which a 2-to-3-point reduction in ESS score is common [11]. Improvements observed in the HNS group in our study showed similar reductions in ESS as seen in the STAR trial [16]. This improvement in sleepiness is an important outcome from a patient’s perspective. The results are relevant for clinical decisions on treatment allocation to patients, especially in the situation of intolerance to certain therapies.

Studies on PAP therapy demonstrated that longer hours of PAP usage per night are associated with greater improvements in daytime sleepiness [32]. However, this effect has never been studied for HNS therapy. The ADHERE registry reported high adherence of 5.6 ± 2.1 h per night with HNS therapy, which is comparable to our study. But, it is unknown whether this effect is dose dependent or not [33]. The usage with HNS was on average approximately one hour longer compared to PAP treatment. This cannot be explained by baseline variables for which the study groups were matched, although we did not control for gender, as probably an influencing factor [34]. Another explanation could be different effects on somnogenic cytokines with HNS treatment compared to PAP therapy [35].

Based on evidence from long-term cohort studies, a reduction of OSA severity can lead to reduced mortality and morbidity and is therefore considered an important treatment goal. Both groups in our study experienced comparable reductions in OSA burden. At month 12 of follow-up, an AHI below 15/h could be detected in each group. This AHI reduction is required for normalization of the cardiovascular risk [36]. Both treatments also meet the target of an AHI ≤ 10 from American Academy for Sleep Medicine (AASM) guidelines for the PAP titration [37]. The observed improvements of OSA severity with both treatments are comparable with previous reports [11].

As in several chronic conditions, it is known for OSA that many patients do not use their prescribed treatment for the total required time. In this case, the total required time would be the total sleep time. We hypothesized that similar outcomes between HNS and PAP therapy would be achieved because the well-known superior efficacy of PAP in reducing OSA severity would be offset by inferior adherence relative to HNS therapy. Indeed, the data on efficacy and adherence from matched cohorts in this study support our hypothesis. Therefore, we calculated the MDA, knowing that most of the OSA participants are not using their treatment during the total sleep time. In our study groups, the usage time was 4.0 h per night for PAP and 5.0 h per night for HNS. When compared to the average sleeping time, which is approximately 6.5 h per night in western industrialized countries, OSA control may not be achieved throughout the entire night in some patients [38]. Consequently, calculation of the MDA is important to fully understand treatment benefits, as it accounts for total sleep time by adjusting treatment efficacy with treatment usage. Of course, the MDA is to be regarded as a concept, whereas the overall clinical data are used while attempting to visualize the group data in terms of the product of adherence and effect of a specific OSA therapy and then to compare this calculated overall clinical effectiveness mutually*.* In this study, the MDA with HNS is superior to PAP therapy, which is related to the increased adherence of approximately 1 h per night. This finding adds an important dimension to the discussion on therapeutic outcomes across the treatment landscape, as MDA allows a more comprehensive assessment than looking at short-term AHI improvements.

### Study limitations

There are several study limitations to our study. First, we did not include randomization between treatment assignments. This is primarily due to ethical concerns of comparing PAP therapy as a first-line treatment for OSA to HNS therapy as a second-line treatment. All patients who received HNS therapy were untreated for an average of 30 months at the time of implantation. A possible allocation to another PAP attempt could have exposed them to additional risks from untreated moderate to severe OSA. Secondarily, the German reimbursement framework does not allow HNS therapy to be used as first-line treatment. Therefore, a priori randomization of patients was not an option. Propensity score matching provides a pragmatic alternative by directly matching patients with comparable clinical parameters by using the nearest-neighbor algorithm.

We also recognize that the HNS group consisted of subjects who failed PAP treatment before receiving stimulation therapy. This may have influenced the adherence since patients who are non-adherent to a first-line treatment and experience subjective improvements with a second-line treatment could have higher adherence to this treatment. It is also not clear what effect the convenience of treatments has on adherence rates. It seems natural that a therapy with greater convenience of use, such as HNS therapy, might have an inherent adherence benefit over treatments where the burden of use is greater.

Another limitation could be that subjects in the HNS group received a DISE before surgery. It is conceivable that some participants in the PAP group might have had complete concentric collapse at the soft palate or other anatomical variants that could contribute to lower PAP efficacy. Since there is no evidence of a lower response to PAP ventilation in patients with complete concentric collapse, we believe this potential bias is negligible. In addition, patients with HNS and PAP had a comparable body mass index slightly below 30 kg/m^2^, and thus, the likelihood of a concentric soft palate collapse should be comparable in both groups too [Steffen A 2015].

Objective outcome assessment was based on HST in both groups, while patients underwent in-lab polysomnography at baseline. We acknowledge that the two methods are difficult to compare with regard to AHI measurement, as type 3 devices carry a systematic underestimation of OSA burden due to lack of EEG recording. As the same diagnostic tools were used in both groups at the two time points, we think the data is sufficiently robust though to allow comparisons between the two treatments. Furthermore, the use of HST for outcome assessment is clinical standard in many healthcare systems and the use of PSG was not possible due to reimbursement constraints. Furthermore, for treatment effectiveness using the AHI from a single night, HST can mislead to a night-to-night variability. Several nights of HST measures would be useful.

Using propensity score matching for statistical analysis is further inherently limited by the risk of selection bias. In fact, although some observed baseline covariates between groups can be balanced, nothing can be done to balance unmeasured characteristics and confounders making the results of propensity score studies sometimes difficult to interpret. We tried to overcome the risk of selection bias by enrolling patients consecutively. Due to too few women presenting in our clinics for OSA treatment, we were not able to match patients for gender, which resulted in more female patients in the PAP group. Nevertheless, this effect was not statistically significant. This unfortunately reflects the worldwide discrepancy with fewer females receiving diagnosis and being treated for OSA, which is also true for HNS therapy [14].

## Conclusion

In this comparative study with matched cohorts, HNS provided greater improvements of sleepiness over PAP therapy in patients with symptomatic OSA. The overall clinical effectiveness, calculated with mean disease alleviation, was superior with HNS therapy compared to PAP ventilation.

## Data Availability

The datasets used and/or analyzed during the current study are available from the corresponding author upon reasonable request.
